# Novel anti-biofouling light-curable fluoride varnish containing 2-methacryloyloxyethyl phosphorylcholine to prevent enamel demineralization

**DOI:** 10.1038/s41598-018-38255-2

**Published:** 2019-02-05

**Authors:** Jae-Sung Kwon, Myung-Jin Lee, Ji-Young Kim, Dohyun Kim, Jeong-Hyun Ryu, Sungil Jang, Kwang-Mahn Kim, Chung-Ju Hwang, Sung-Hwan Choi

**Affiliations:** 1 Department and Research Institute of Dental Biomaterials and Bioengineering, Yonsei University College of Dentistry, Seoul, Republic of Korea; 2 Department of Orthodontics, Institute of Craniofacial Deformity, Yonsei University College of Dentistry, Seoul, Republic of Korea; 3 Department of Conservative Dentistry, Yonsei University College of Dentistry, Seoul, Republic of Korea; 4 BK21 PLUS Project, Yonsei University College of Dentistry, Seoul, Republic of Korea; 5 Department of Oral Biology, Yonsei University College of Dentistry, Seoul, Republic of Korea

## Abstract

We evaluated the efficacy of light-curable fluoride varnish (LCFV) that contains 2-methacryloyloxyethyl phosphorylcholine (MPC) in terms of anti-biofouling properties and prevention of tooth enamel demineralization. MPC was mixed with and incorporated into LCFV at 0 (control), 1.5, 3.0, 5.0, 10.0, 20.0, and 40.0 weight percentage (wt%). Addition of high wt% of MPC resulted in increased film thickness and decreased the degree of conversion, indicating loss of the advantageous properties of LCFV. Addition of 1.5, 3, or 5 wt% MPC significantly reduced the amount of bovine serum albumin adsorbed from a solution and proteins adsorbed from brain heart infusion medium compared to the control (*P* < 0.001). A similar pattern was observed for bacterial adhesion: significantly less *Streptococcus mutans* cells adhered on the surface of LCFV with 1.5, 3, or 5 wt% MPC (*P* < 0.001) than on the control, and similar results were obtained for *Actinomyces naeslundii* and *Streptococcus sanguinis* adherence to LCFV with 3 wt% MPC. Finally, bacterial adhesion, surface microhardness loss, and the depth of demineralization were substantially lower on bovine tooth enamel surface coated with LCFV containing 3 wt% of MPC than in the control treatment (0 wt% MPC). Therefore, this novel LCFV containing a low concentration of MPC (e.g., 3 wt%) would be effective in anti-biofouling while maintaining the important advantageous features of light-curable fluoride in preventing demineralization.

## Introduction

Caries or white spots are highly prevalent in the permanent teeth of children and adolescents who do not regularly brush their teeth, especially when brushing is hampered by factors such as orthodontic fixed appliances^1,2^. These lesions are mainly due to the demineralization of hard tissue by lactic acid produced in deposits of bacterial biofilm, including saliva proteins and food residues that are not removed from the tooth surface by brushing^3^. To minimize such erosive tooth loss, several preventive strategies have been employed, among which fluoride application has been popular choice^4^.

Topical fluoride applications in the form of fluoride varnish have been used extensively and proven effective in the prevention of demineralization^1,5^; however, repeated application is required for conventional fluoride varnish to retain its anti-caries effect^6^. Hence, light-curable fluoride varnish (LCFV), which has been shown to be advantageous in terms of longevity and sustainability^7,8^, has been increasingly used. Several studies have shown that LCFV effectively prevents enamel demineralization on a longer term than conventional fluoride varnish^7,8^. Moreover, the occurrence of white spot lesions during comprehensive orthodontic treatment can be prevented by the use of LCFV^9^. Still, fluoride varnish has a limitation in that it does not fully protect the underlying dental tissue, while fluoride alone cannot effectively prevent enamel demineralization^10,11^.

Zwitterionic materials are a group of materials that possess both anionic and cationic groups such that their overall charge is neutral^12^. Because of these properties, these materials have superior anti-biofouling effects. One of the most commonly used zwitterionic materials is 2-methacryloyloxyethyl phosphorylcholine (MPC), which is a methacrylate that harbours a phospholipid polar group in the side chain, providing a highly hydrophilic surface that can resist protein absorption and bacterial adhesion^13^. Various biomaterials based on MPC polymers have been investigated^13,14^, and recent studies have attempted to apply this material in dentistry, e.g., through incorporation into composite materials^15,16^, in orthodontic cements^17^, and in dentin bonding agents^18,19^. However, no study has been reported on the incorporation of MPC in fluoride varnishes for the prevention of dental caries. If MPC can be successfully applied in fluoride varnishes, especially durable LCFV, it will be possible to effectively prevent dental plaque formation and dental caries including white spots, especially in high-risk children and adolescents.

Therefore, in the current study, we attempted to synthesize LCFV containing MPC with the aim to combine the anti-biofouling activity of MPC with the important enamel demineralization-preventive feature of LCFV, without impairing the critical mechanical properties of LCFVs. Using experiments *in vitro* and *ex vivo*, we evaluated the protein-repellent and bacterial adhesion-preventive properties of MPC-incorporated LCFV. In addition, the clinical application of the material was evaluated by assessing its effect in terms of prevention of enamel demineralization in bovine teeth. We hypothesized that the combination of MPC and LCFV would achieve superior protein-repellent effect, prevention of bacterial adhesion, and prevention of enamel demineralization, compared to LCFV without MPC.

## Methods

### Preparation of MPC-incorporated LCFV

Commercially available MPC powder (Sigma-Aldrich, St. Louis, MO, USA) and LCFV (Clinpro XT Varnish; 3 M ESPE, St. Paul, MN, USA) were used in this study. MPC powder was mixed into LCFV at various weight percentages (1.5, 3.0, 5.0, 10.0, 20.0, and 40.0 wt%), while LCFV without MPC was used as a control. The compositions of the experimental and control materials are summarized in Table 1. To evaluate the degree of conversion (DC), protein adsorption, and bacterial analyses, prepared samples were placed in a mould with diameter of 15 mm and a thickness of 2 mm to form disc-shaped specimens. To assess film thickness and for application to bovine teeth, samples were placed in a syringe and 0.05 mL of sample was ejected on glass plate or bovine tooth, respectively. All samples were then polymerized using a LED light-curing unit (Elipar S10; 3 M ESPE Co., Seefeld, Germany).

**Table 1:** Compositions of the tested materials.

Groups	Light-Curable Fluoride Varnish (LCFV, Clinpro XT Varnish), wt%	2-Methacryloyloxyethyl Phosphorylcholine (MPC), wt%
Control	100	0
1.5% MPC	98.5	1.5
3% MPC	97.0	3.0
5% MPC	95.0	5.0
10% MPC	90.0	10.0
20% MPC	80.0	20.0
40% MPC	60.0	40.0

### Degree of conversion

The DC was evaluated by Fourier-transform infrared spectroscopy (FTIR; Vertex 70; Bruker Optik, Ettlingen, Germany). The spectrometer was coupled to a horizontal attenuated total reflectance (ATR) device consisting of a 2-mm-diameter diamond crystal (Platinum ATR-QL; Bruker Optik, Baden-Württemberg, Germany). The diameter of the measured surface was 800 µm, the wave number range of the spectrum was 1400–2000 cm^−1^, and the FTIR spectra were recorded at a rate of 2 scans per second and at a resolution of 4 cm^−1^. To determine the percentage of unreacted double bonds, the DC was assessed as the variation of the absorbance intensities’ peak area ratio of the methacrylate carbon double bond (peak 1634 cm^−1^) and those of an internal standard (aromatic carbon double bond; peak at 1608 cm^−1^) during polymerization, in relation to the uncured material^20^.

### Protein adsorption

Protein adsorption was tested out according to a previously established method^19^. Briefly, all disc-shaped samples were immersed in fresh phosphate-buffered saline (PBS; Gibco, Grand Island, NY, USA) at room temperature for 1 h. Then, each sample was immersed in a protein solution of either bovine serum albumin (BSA; Pierce Biotechnology, Rockford, IL, USA) or brain heart infusion (BHI; Difco, Sparks, MD, USA) broth, both at a concentration of 2 mg of protein per mL of PBS and a volume of 100 μL. After 4 h of incubation under sterile humid conditions at 37 °C in 5% CO_2_, any non-adherent protein was removed by washing twice with PBS. The adherent protein was then reacted with 200 μL of micro-bicinchoninic acid (Micro BCA^TM^ Protein Assay Kit; Pierce Biotechnology) followed by incubation at 37 °C for 30 min. The amount of adsorbed protein was quantified by measuring the absorbance at 562 nm using a micro-plate reader (Epoch; BioTek Instruments, VT, USA).

### Bacterial analyses

Bacterial analyses were carried out using *Streptococcus mutans* (ATCC 25175), *Streptococcus sanguinis* (ATCC 10556), and *Actinomyces naeslundii* (KCOM 1942; Korean Collection for Oral Microbiology (KCOM, Gwangju, Korea)). Streptococci were cultured in Brain Heart Infusion broth (Becton Dickinson and Co., Sparks, MD, USA). *A. naeslundii* was cultured in BHI broth supplemented with 0.5% yeast extract (Becton Dickinson and Co.), 0.0001% resazurin (Sigma-Aldrich, St. Louis, MO, USA), 0.05% Hemin (Sigma-Aldrich), 0.05% of cysteine (Sigma-Aldrich) and 0.02% vitamin K (Sigma-Aldrich) under anaerobic condition in an Anaeropack (Mitsubishi Gas Chemical, Tokyo, Japan).

Following the preparation of disc-shaped specimens, 1 mL of bacterial suspension (1 × 10^8^ cells/mL) was placed on each disc in a 24-well plate and incubated at 37 °C for 24 h. After incubation, the samples were gently washed twice with PBS to remove any non-adherent bacteria.

For microscopic examination of attached bacteria, bacteria on the samples were fixed with 2% glutaraldehyde-paraformaldehyde in 0.1 M PBS for at least 30 min, at room temperature. The samples were post-fixed with 1% OsO_4_ dissolved in 0.1 M PBS for 2 h, dehydrated in an ascending gradual series of ethanol, treated with isoamyl acetate, and subjected to critical point drying (LEICA EM CPD300; Leica, Wien, Austria). Then, the discs were coated with Pt (5 nm) by using an ion coater (ACE600; Leica) and examined and photographed using a scanning electron microscopy (FE-SEM; Merin, Carl Zeiss, Oberkochen, Germany) at 2 kV.

To evaluate bacterial colony forming units (CFU), adherent bacteria were harvested in 1 mL BHI by sonication (SH-2100; Saehan Ultrasonic, Seoul, Korea) for 5 min. Of this bacterial suspension, 100 µL was spread onto an agar plate and incubated at 37 °C for 24 h. Then, the total number of colonies was counted.

The viability of adherent bacteria was examined by staining using a live/dead bacterial viability kit (Molecular Probes, Eugene, OR, USA), according to the manufacturer’s protocols. Equal volumes of Syto 9 dye and propidium iodide, which stain live and dead bacteria, respectively, from the kit were mixed thoroughly. Of the mixture, 3 µL was added to 1 mL of bacterial suspension prepared as described above. After 15 min of incubation at room temperature in the dark, the stained samples were observed under a confocal laser microscope (LSM700; Carl Zeiss, Thornwood, NY, USA). Live bacteria appeared green while dead bacteria appeared red.

### Application to bovine tooth and evaluation of enamel surface demineralization

The experiment was conducted according to a previously established method^21^, with modifications. Following the fixation of a bovine tooth in a resin block, the surface was polished to expose the enamel. After etching the enamel with 20% phosphoric acid (Reliance Ortho Prod. Inc., IL, USA) for 15 s and rinsing with water, 0% MPC-LCFV (control) and 3% MPC-LCFV was applied to each half of the exposed enamel followed by light curing. Coated bovine teeth were then placed in each well of a 24-well plate and exposed to BHI culture medium supplemented with 2% sucrose (1,492.5 μL/well) and 7.5 μL of *S. mutans* inoculum (~1 × 10^8^ cells/mL). The plate was incubated at 37 °C for 48 h to simulate enamel demineralization by the acid produced by the bacteria^21^. The bovine tooth was stained with Trace Disclosing Solution (Young Dental Manufacturing, MO, USA) and examined under FE-SEM using the procedures described above. In addition, the percentage of Vickers microhardness loss of each sample was measured, using a micro-indentation hardness tester (DMH-2; Matsuzawa Seiki, Japan), in compliance with the following expression: 100 × (*initial surface microhardness value – final surface microhardness value)/initial surface microhardness value*, where the ‘initial and final surface microhardness values’ were the surface microhardness values before and after immersion into the bacterial solution, as an indication of enamel demineralization.

### Demineralization study by polarized light microscopy

Demineralization of bovine teeth following application of 0% MPC-LCFV (control) and 3% MPC-LCFV was analysed as reported^22^, with some modification. First, bovine teeth were cut in slices of 100–150 µm thickness using a low-speed diamond wheel saw (Diamond Cutter RB205 Metsaw, R&B Co., Daejeon, Korea). Then, we applied a commercial surface sealant/polish (BisCover, Bisco Dental, Schaumburg, IL, USA) on the buccolingual area of the sectioned specimen. The enamel surfaces were then etched with 20% phosphoric acid for 15 s and rinsed with water, followed by the application of 0% MPC-LCFV (control) and 3% MPC-LCFV with light curing. Specimens without the application of any varnish were also included. Bovine teeth specimens with or without LCFV were placed in each well of a 24-well plate and were exposed to BHI medium supplemented with 2% sucrose (1492.5 μL/well) and 7.5 μL of *S. mutans* inoculum (~1 × 10^8^ cells/mL). The plate was incubated at 37 °C for 14 days to simulate enamel demineralization by the acid produced by the bacteria^21^. The growth medium was replaced every 48 h to support biofilm regrowth. All samples were observed under a polarized light microscope (Olympus BX41, Olympus, Tokyo, Japan) at a magnification of 40 × before and after demineralization. Images were captured with a camera mounted on the microscope and were analysed using Leica Application Suite, version 4.12.0 (Leica Microsystems, Switzerland)

### Statistical analysis

For all statistical analyses, IBM SPSS software, version 23.0 (IBM Korea Inc., Seoul, Korea) for Windows was used, with data from at least three independent experiments. The results obtained for the control and experimental materials were analysed by one-way analysis of variance (ANOVA) followed by Tukey’s test. Two groups were compared using the independent *t* test. *P* < 0.05 was considered statistically significant.

## Results

### Film thickness and degree of conversion

**Figure 1:**
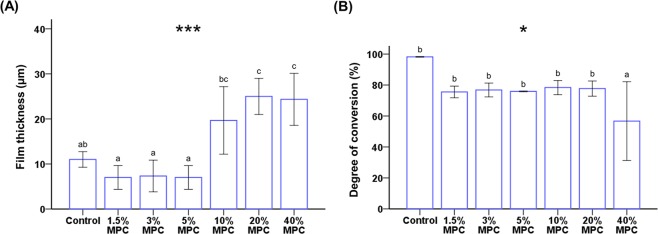
Comparison of the mean film thickness (**A**) and degree of conversion (**B**) between groups. Different letters above bars indicate significant differences. *P < 0.05, ***P < 0.001 for comparisons between LCFV with different concentrations of MPC.

The film thickness of 0–5% MPC-LCFV was 7–11 µm, whereas that of 20% and 40% MPC-LCFV was more than double the thickness of the control (0% MPC-LCFV) (*P* < 0.001, Fig. 1A). In terms of DC, except for 40% MPC, none of the materials showed a significant difference. 40% MPC-LCFV showed a 42% decrease in DC compared to the control (*P* = 0.014, Fig. 1B). Therefore, 40% MPC-LCFV was not used in further experiments.

### Protein adsorption

**Figure 2:**
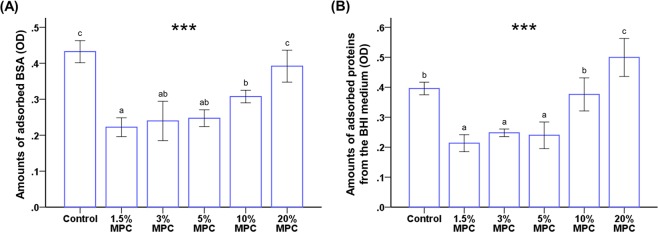
Comparison of the optical density (OD) of adsorbed bovine serum albumin (BSA) (**A**) and protein adsorbed form brain heart infusion (BHI) medium (**B**) between LCFV with different concentrations of MPC. Different letters above bars indicate significant differences. ***P < 0.001 for comparisons between LCFV with different concentrations of MPC.

The amount of adsorbed BSA was significantly lower on 1.5%, 3%, 5%, and 10% MPC-LCFV (range, 0.22–0.30) than on the control LCFV and 20% MPC-LCFV (range, 0.39–0.43) (*P* < 0.001, Fig. 2A). Figure 2B shows that the amount of proteins adsorbed from BHI medium was significantly lower in 1.5%, 3%, and 5% MPC-LCFV (range, 0.21–0.24) than in the control group (0.39 ± 0.02) (*P* < 0.001). There was no significant difference between control and 10% MPC-LCFV (0.37 ± 0.05), whereas 20% MPC-LCFV (0.50 ± 0.06) showed significantly higher protein adsorption. Therefore, 20% MPC-LCFV was not used in further experiments.

### Bacterial analyses

**Figure 3:**
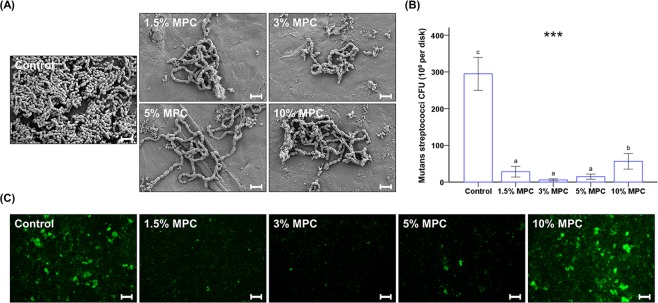
Qualitative scanning electron images of *S. mutans* cells attached to the surfaces of control and experimental groups at a magnification of 5,000× (**A**). Scale bar is 2 µm. Colony-forming unit (CFU) counts derived from *S. mutans* cells attached on the surfaces of control and MPC-LCFV (**B**). Different letters above bars indicate significant differences. ***P < 0.001 for comparisons between LCFV with different concentrations of MPC. Representative live/dead staining images of *S. mutans* cells attached on the surfaces of control and experimental groups (**C**). Scale bar is 100 µm.

FE-SEM images clearly indicated that less *S. mutans* cells adhered to the surface of 1.5%, 3%, 5%, and 10% MPC-LCFV than on the control (Fig. 3A). When analysed quantitatively, 1.5–10% MPC-LCFV showed significantly lower CFU counts than the control (*P* < 0.001, Fig. 3B). Further, the CFU counts for 1.5%, 3%, and 5% MPC-LCFV were significantly lower than that for 10% MPC-LCFV and were about 1/20 of the control. There was no statistically significant difference between the 1.5%, 3%, and 5% MPC-LCFV, but 3% MPC-LCFV showed the lowest count. These findings were confirmed by viability staining results, where less live bacteria (visible as green in Fig. 3) were attached to 3% MPC-LCFV than on the control (Fig. 3C). However, there was no evidence of dead bacteria (visible as red in Fig. 3) on both control and 3% MPC-LCFV.

**Figure 4:**
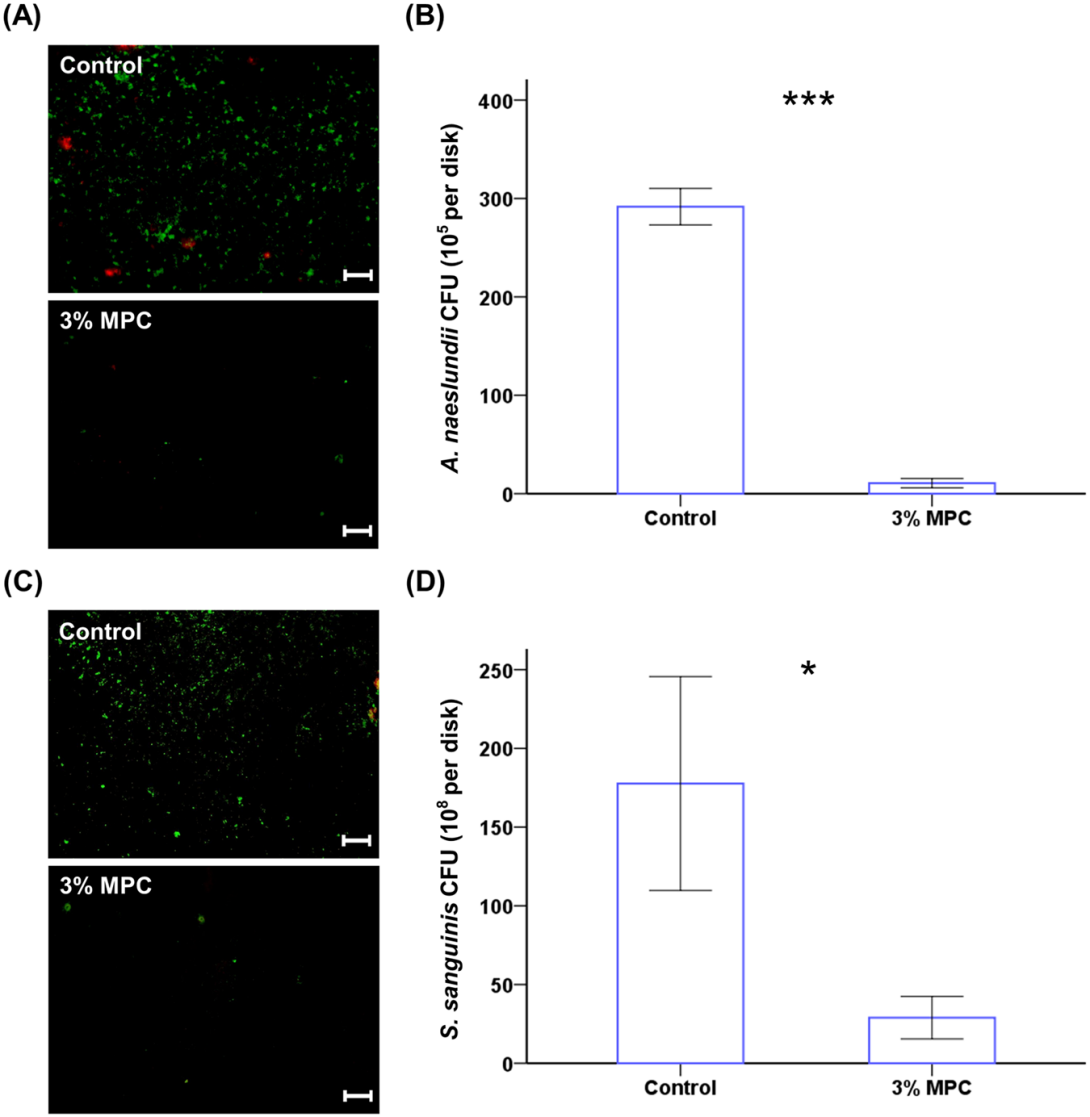
Representative live/dead staining images of *A. naeslundii* (**A**) and *S. sanguinis* (**C**) cells attached on the surfaces of control and 3% MPC-LCFV. Scale bar is 100 µm. CFU counts derived from *A. naeslundii* (**B**) and *S. sanguinis* (**D**) cells attached on the surfaces of control and 3% MPC-LCFV. *P < 0.05, ***P < 0.001 for comparisons between LCFV with 3% MPC-LCFV.

Similar results were obtained for *A. naeslundii* and *S. sanguinis*. The CFU counts for 3% MPC-LCFV were significantly lower than that in the control (Fig. 4B,D, *P* < 0.001 and *P* = 0.021, respectively), and viability staining revealed that less live bacteria were attached to 3% MPC-LCFV than on the control for both species (Fig. 4A,C). We observed some dead *A. naeslundii* and *S. sanguinis* cells on the control, while no dead bacteria were detected on 3% MPC-LCFV (Fig. 4A,C).

### Application to bovine tooth and evaluation of enamel surface microhardness loss

**Figure 5:**
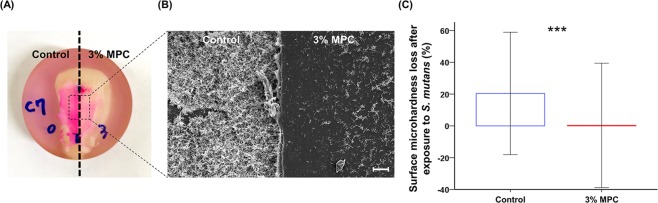
Representative photograph of a bovine tooth to which control and 3% MPC-LCFV was applied on each side and that was exposed to BHI culture medium supplemented with 2% sucrose and bacterial inoculum. Trace Disclosing Solution was applied to the sample (**A**). Representative scanning electron image of bacteria attached on the surfaces of the control and 3% MPC-LCFV at a magnification of 100× (**B**). Scale bar is 40 µm. Comparison of enamel surface microhardness loss (%) after exposure to a bacterial culture between the control and 3% MPC-LCFV (**C**). ***P < 0.001 for comparisons between the control and 3% MPC-LCFV before and after exposure a bacterial culture.

To evaluate the clinical application potential of LCFV containing MPC, 0% and 3% MPC-LCFV was applied on each side of a bovine tooth, which was then exposed to appropriate culture medium containing bacteria for 48 h. The application of Trace Disclosing Solution resulted in bright red coloration on the side where control LCFV was applied (left), while a faded red coloration was evident on the side where 3% MPC-LCFV was applied (right) (Fig. 5A). This was confirmed by FE-SEM (Fig. 5B), where a clear difference in the number of bacteria adherent to the surface was evident between the two sides. The Vickers microhardness showed no significant difference between control and 3% MPC-LCFV before exposure to a bacterial solution. However, the hardness loss following exposure to the bacterial solution was significantly (*P* < 0.001) higher for control LCFV (20.38 ± 38.47%) than for 3% MPC-LCFV (0.30 ± 39.08%) (Fig. 5C).

### Demineralization study by polarized light microscopy

**Figure 6:**
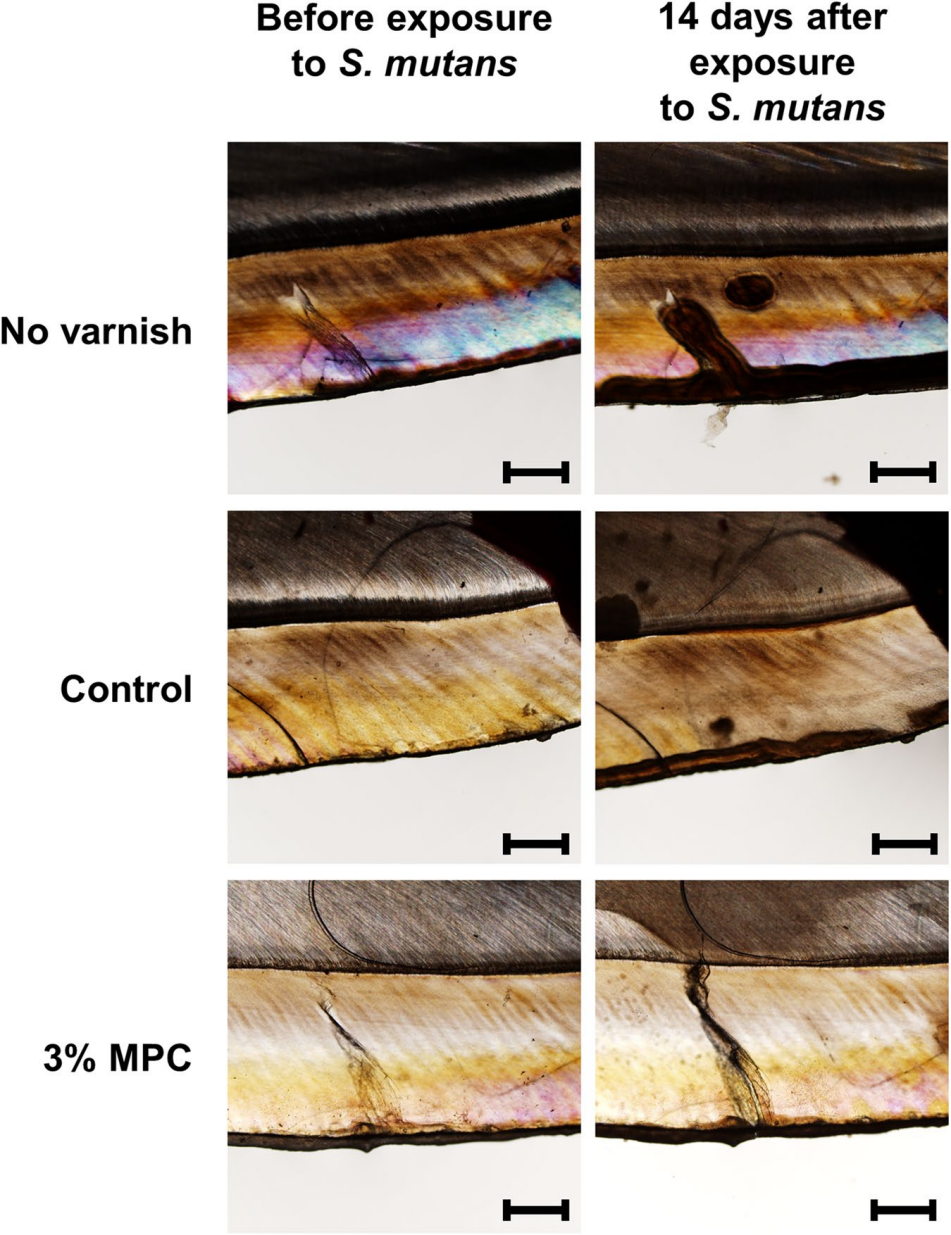
Representative polarized light microscopy images of a bovine tooth before and 14 days after exposure to BHI culture medium supplemented with 2% sucrose and bacterial inoculum. Specimens were treated with 0% MPC-LCFV (control), 3% MPC-LCFV, or no varnish. Scale bar is 500 µm.

Following 14 days of immersion in acidic solution resulting from the fermentation of sucrose by *S. mutans*, specimens without varnish showed dramatic demineralization of the enamel as indicated by the presence of dark brown areas. However, 3% MPC-LCFV effectively protected against demineralization, whereas 0% MPC-LCFV (control) showed a slight increase in the area of demineralized tissue as compared to 3% MPC-LCFV (Fig. 6). Image analysis revealed that the average depth of demineralization was significantly lower for 0% MPC-LCFV (46.44 ± 44.88 μm) and 3% MPC-LCFV (23.62 ± 23.03 μm) than for the sample without varnish (260.46 ± 97.51 μm) (*P* < 0.001).

## Discussion

To date, in the dental field, MPC has been applied mainly to composite materials^15,16,23^, in orthodontic cements^17^, and in dentin bonding agents^18,19^, alone or in combination with antibacterial materials, to block the attachment of bacteria to the resin and to kill already-deposited bacteria, mainly to resist recurrent caries caused by micro-leakage of tooth and resin restorations. However, application of zwitterionic materials such as MPC in fluoride varnishes as in the present study would allow primary prevention of dental caries in high-caries-risk children and adolescents. To our knowledge, this study is the first to investigate the effects of MPC incorporation into LCFV on anti-biofouling and enamel demineralization-inhibitory effects. Moreover, to ensure clinical usefulness of the results of this study, using experiments *in vitro* and *ex vivo*, we sought to find optimal MPC content ratios that allowed maximum anti-biofouling effects without impairing the critical mechanical properties of LCFVs.

The effectiveness and advantages of LCFV have been shown in various previous studies^7–9^, and to maintain these advantages upon addition of MPC, two important factors determining LCFV effectiveness were considered; film thickness and DC. It was evident that 20% MPC-LCFV had a film thickness more than twice that of the control LCFV, while the degree of polymerization was decreased by 42% for 40% MPC-LCFV when compared with the control. Therefore, it was evident that such high MPC contents would cause discomfort in the patient and may require a higher number of varnish applications, as polymerization is inhibited^7^.

Previous studies have demonstrated that the addition of MPC results in biocompatible and hydrophilic biomedical polymers, and hydrophilic material surfaces have been shown to be more resistant to protein absorption than hydrophobic surfaces^14^. In this study, we assessed protein adsorption of BSA, which is considered a protein with a quite general structure, and a mix of proteins that are present in bacterial BHI culture medium. First, it was noted that the amount of adsorbed BSA was significantly lower for 1.5%, 3%, 5%, and 10% MPC-LCFV than for the control and 20% MPC-LCFV. A similar pattern was observed for the amount of proteins adsorbed from BHI medium. MPC is a methacrylate with a phospholipid polar group in the side chain^14^. Phospholipid molecules generally consist of hydrophilic heads that are attracted to water and hydrophobic tails that are repelled by water^24^. Thus, in water, the MPC phospholipids will orient themselves into a bilayer in which the non-polar tails face the inner area of the bilayer and the polar heads face outward to interact with the water, which results in its highly hydrophilic properties^14^. When MPC polymer is exposed to a protein solution, the unique structure of MPC would allow a large amount of free water to be present around the phosphorylcholine group, whereas there would be no bound water in the hydrated MPC^13^. As the presence of bound water would cause protein adsorption, whereas the presence of free water would repel protein absorption, the addition of MPC to LCFV results in protein-repellent properties^25^. Previous studies have demonstrated that copolymerization of MPC with resin-like polymers resulted in MPC immobilization and caused long-lasting and durable prevention of protein attachment^26,27^. However, high contents of MPC, such as 20%, resulted in significantly higher protein adsorption in our study. Such a phenomenon has been also reported in previous studies that considered the addition of MPC into dental composite resin^28^, polymethyl methacrylate^26^, and polyethylene^29^. It has been reported that despite the increase in protein-repellent efficacy as MPC concentration increased, the entire polymerization system began to show gelation at higher concentrations of MPC, which resulted in marked decrease in protein-repellent efficacy^26,29^.

Dental plaque is formed when microorganisms aggregate on the tooth surface^30^. The initial step in this process is the absorption of salivary-derived proteins as a salivary pellicle that can mediate bacterial attachment and biofilm formation^31^. Hence, the protein-repellent properties of MPC-incorporated LCFV would result in resistance to bacterial adhesion, which were analysed using three different bacterial sepcies *S. mutans*, *A. naeslundii* and *S. sanguinis*, which are pathogenic bacteria commonly found in the oral cavity and involved in the early stage of oral biofilm formation^32,33^. Indeed, less bacteria adhered on the surfaces of all MPC-LCFV than on control LCFV as indicated by microscopy and CFU counting, for all three species. For *S. mutans*, the result was consistent with the findings on protein adsorption, as the CFU counts increased with the incorporation of 10% MPC. Finally, it was evident that bacteria were prevented from attaching to the surface rather than being killed on the surface, as there was no evidence of dead bacteria on 3% MPC-LCFV. As dead bacteria were not detected on the surfaces treated with LCFV containing MPC in the live/dead cell assay, the MPC anti-biofouling effect seems to mainly depend on nonspecific protein repellence, not on antimicrobial properties. This implies that the risk for toxic effects on normal tissues in the oral cavity is low and that zwitterionic materials such as MPC can be safely applied in children and adolescents. Incorporation of antibacterial agents such as chlorhexidine into the material may lead to toxic effects or induce population shifts in the oral microbiota^23^.

When we evaluated the clinical application potential of MPC-LCFV, both the Trace Disclosing Solution assay and FE-SEM clearly indicated that less bacteria adhered to 3% MPC-LCFV than to the control-treated enamel after 48 h of exposure to a bacterial culture. More importantly, surface microhardness loss was significantly increased for control-treated enamel, whereas enamel coated with 3% MPC-LCFV maintained microhardness after exposure to the bacterial culture. This result was confirmed by assessing demineralization by polarized light microscopy. LCFV itself has a demineralization-preventive effect as indicated by previous studies^7–9^. However, the effect was improved with addition of MPC into LCFV, as the depth of demineralized tissue was smaller on 3% MPC-LCFV than on 0% MPC-LCFV. It has been suggested that, despite the advantages of fluoride varnishes, they have low acid resistance and consequently do not fully protect underlying dental tissues against demineralization^11^. In the current study, demineralization was assessed only for 14 days, and in artificial environment. It can be expected that under longer demineralization caused by a highly acidic environment, the difference between 3% and 0% MPC-LCFV might become even more pronounced. Further, the use of fluoride varnish alone cannot prevent enamel demineralization^10^. This study clearly indicated that the addition of an appropriate amount of MPC to fluoride varnish results in a synergetic effect in terms of bacterial repellent activity and consequent prevention of demineralization.

As we used *in-vitro* and *ex-vivo* experiments, complications in the oral environment, including salivary flow and the presence of food debris, were not evaluated and should be considered in future *in-vivo* or clinical studies. However, the present study showed that the addition of an appropriate amount of MPC into LCFV resulted in protein-repellent properties that consequently reduced bacterial attachment. The addition of 3 wt% of MPC polymer was shown to be optimal in terms of both providing anti-biofouling properties and maintaining the advantageous features of LCFV. Finally, the application of LCFV with 3 wt% MPC polymer effectively prevented enamel demineralization in a bovine tooth model.

## References

[1] Marinho, V. C., Worthington, H. V., Walsh, T. & Clarkson, J. E. Fluoride varnishes for preventing dental caries in children and adolescents. *Cochrane Database Syst Re*v. doi:10.1002/14651858.CD002279.pub2.CD002279 (2013). 10.1002/14651858.CD002279.pub2 PMC10758998 23846772

[2] Salas MM, Nascimento GG, Huysmans MC, Demarco FF (2015). Estimated prevalence of erosive tooth wear in permanent teeth of children and adolescents: an epidemiological systematic review and meta-regression analysis. J Dent.

[3] Cenci MS, Pereira-Cenci T, Cury JA, Ten Cate JM (2009). Relationship between gap size and dentine secondary caries formation assessed in a microcosm biofilm model. Caries Res.

[4] Lussi A, Carvalho TS (2015). The future of fluorides and other protective agents in erosion prevention. Caries Res.

[5] Chestnutt IG (2017). Fissure Seal or Fluoride Varnish? A Randomized Trial of Relative Effectiveness. J Dent Res.

[6] Petersson LG (1991). Caries-inhibiting effects of different modes of Duraphat varnish reapplication: a 3-year radiographic study. Caries Res.

[7] Mehta A (2015). Effect of light-curable fluoride varnish on enamel demineralization adjacent to orthodontic brackets: an *in-vivo* study. Am J Orthod Dentofacial Orthop.

[8] Zhou SL (2012). *In vitro* study of the effects of fluoride-releasing dental materials on remineralization in an enamel erosion model. J Dent.

[9] Kumar Jena A, Pal Singh S, Kumar Utreja A (2015). Efficacy of resin-modified glass ionomer cement varnish in the prevention of white spot lesions during comprehensive orthodontic treatment: a split-mouth study. J Orthod.

[10] Chapman JA (2010). Risk factors for incidence and severity of white spot lesions during treatment with fixed orthodontic appliances. Am J Orthod Dentofacial Orthop.

[11] Ganss C, Schlueter N, Klimek J (2007). Retention of KOH-soluble fluoride on enamel and dentine under erosive conditions–A comparison of *in vitro* and *in situ* results. Arch Oral Biol.

[12] Jiang S, Cao Z (2010). Ultralow-fouling, functionalizable, and hydrolyzable zwitterionic materials and their derivatives for biological applications. Adv Mater.

[13] Moro T (2009). Wear resistance of artificial hip joints with poly(2-methacryloyloxyethyl phosphorylcholine) grafted polyethylene: Comparisons with the effect of polyethylene cross-linking and ceramic femoral heads. Biomaterials.

[14] Lewis AL, Tolhurst LA, Stratford PW (2002). Analysis of a phosphorylcholine-based polymer coating on a coronary stent pre- and post-implantation. Biomaterials.

[15] Zhang N (2015). A novel protein-repellent dental composite containing 2-methacryloyloxyethyl phosphorylcholine. Int J Oral Sci.

[16] Zhang N (2015). Protein-repellent and antibacterial dental composite to inhibit biofilms and caries. J Dent.

[17] Zhang N (2016). Orthodontic cement with protein-repellent and antibacterial properties and the release of calcium and phosphate ions. J Dent.

[18] Zhang N, Melo MA, Bai Y, Xu HH (2014). Novel protein-repellent dental adhesive containing 2-methacryloyloxyethyl phosphorylcholine. J Dent.

[19] Zhang N (2015). Development of a multifunctional adhesive system for prevention of root caries and secondary caries. Dent Mater.

[20] Lee, M. J. *et al*. Cytotoxicity of Light-Cured Dental Materials according to Different Sample Preparation Methods. *Materials* **10** (2017). 10.3390/ma10030288 PMC5503327 28772647

[21] Vieira TI (2018). Cytotoxicity of novel fluoride solutions and their influence on mineral loss from enamel exposed to a Streptococcus mutans biofilm. Archives of Oral Biology.

[22] Medeiros MID (2018). TiF4 varnish protects the retention of brackets to enamel after *in vitro* mild erosive challenge. J Appl Oral Sci.

[23] Imazato S (1994). Incorporation of Bacterial Inhibitor into Resin Composite. Journal of Dental Research.

[24] Busscher HJ, Rinastiti M, Siswomihardjo W, van der Mei HC (2010). Biofilm formation on dental restorative and implant materials. J Dent Res.

[25] Hori K, Matsumoto S (2010). Bacterial adhesion: From mechanism to control. Biochemical Engineering Journal.

[26] Exterkate RA, Crielaard W, Ten Cate JM (2010). Different response to amine fluoride by Streptococcus mutans and polymicrobial biofilms in a novel high-throughput active attachment model. Caries Res.

[27] Xu X (2012). Synthesis and characterization of antibacterial dental monomers and composites. J Biomed Mater Res B Appl Biomater.

[28] Weng Y (2012). A novel antibacterial resin composite for improved dental restoratives. J Mater Sci Mater Med.

[29] Shinohara MS (2009). Fluoride-containing adhesive: durability on dentin bonding. Dent Mater.

[30] Katsikogianni M, Missirlis YF (2004). Concise review of mechanisms of bacterial adhesion to biomaterials and of techniques used in estimating bacteria-material interactions. Eur Cell Mater.

[31] Pratt-Terpstra IH, Weerkamp AH, Busscher HJ (1989). The effects of pellicle formation on streptococcal adhesion to human enamel and artificial substrata with various surface free-energies. J Dent Res.

[32] Nedeljkovic I (2017). Biofilm-induced changes to the composite surface. J Dent.

[33] Terefework Z (2008). MLPA diagnostics of complex microbial communities: relative quantification of bacterial species in oral biofilms. J Microbiol Methods.

